# Supplementary material for: Empirical methods for controlling false positives and estimating confidence in ChIP-Seq peaks
Source: BMC Bioinformatics. 2008 Dec 5;9:523. doi: 10.1186/1471-2105-9-523 (PMC2628906; doi:10.1186/1471-2105-9-523)
Supplement: Additional File 1 — A variety of html documents from the USeq web site detailing the available applications, their best usage, output file type descriptions, command line menus, etc. [file 1471-2105-9-523-S1.zip › USeqUserGuides/JavaDoc.zip!/JavaDoc/util/gen/Job.html]

Job


|  |  |  |
| --- | --- | --- |
| **Overview** \| **Package** \| **Class** \| **Use** \| **Tree** \| **Deprecated** \| **Index** \| **Help** | |  |
| **PREV CLASS**   **NEXT CLASS** | **FRAMES**    **NO FRAMES**     **All Classes** |  |
| SUMMARY: NESTED \| FIELD \| CONSTR \| METHOD | DETAIL: FIELD \| CONSTR \| METHOD |  |


---


## util.gen Class Job

```
java.lang.Object
  util.gen.Job
```

---

``` public class Job extends java.lang.Object ```

---

**Constructor Summary**

`Job(java.lang.String[] lines)`


| **Method Summary** | |
| --- | --- |
| `static java.util.HashMap` | `loadHash(java.lang.String[] lines)`             Converts a String[] of key = values into a HashMap. |
| `static java.lang.String` | `parseJobId(java.lang.String[] lines)`             Attempts to parse 'Job Id:', returns null if not found. |

**Methods inherited from class java.lang.Object**

`equals, getClass, hashCode, notify, notifyAll, toString, wait, wait, wait`

**Constructor Detail**

### Job

```
public Job(java.lang.String[] lines)
```


**Method Detail**

### loadHash

```
public static java.util.HashMap loadHash(java.lang.String[] lines)
```

:   Converts a String[] of key = values into a HashMap. Skips lines that don't
    contain a ' = '.

---


### parseJobId

```
public static java.lang.String parseJobId(java.lang.String[] lines)
```

:   Attempts to parse 'Job Id:', returns null if not found.


---


|  |  |  |
| --- | --- | --- |
| **Overview** \| **Package** \| **Class** \| **Use** \| **Tree** \| **Deprecated** \| **Index** \| **Help** | |  |
| **PREV CLASS**   **NEXT CLASS** | **FRAMES**    **NO FRAMES**     **All Classes** |  |
| SUMMARY: NESTED \| FIELD \| CONSTR \| METHOD | DETAIL: FIELD \| CONSTR \| METHOD |  |


---
